# On the Transformation of the Encysted Entozoa into Tape-Worms

**Published:** 1853-02

**Authors:** Jno. C. Dalton


ART. II.—*On the Transformation of the Encysted Entozoa into Tape-Worms.* By JNO. C. DALTON, Jr., M. D.

The following report of some experiments by Prof. C. Th. von Siebold, will be found of considerable importance, not only from their general zoological interest, but more particularly from their bearing on the question of spontaneous generation. Notwithstanding that the idea of spontaneous or equivocal generation is repudiated by the great majority of physiologists at the present day, it seems to be still entertained in some quarters, even by men of considerable scientific attainments. The defences of this theory, however, have one after another given way before the advance of zoological science. It was demonstrated long ago by Redi and Valisnieri, that the worms, which appear in putrefying flesh, were not produced, as previously supposed, from the decomposing animal matter itself, but were the larvæ of winged insects, which had been led, by a curious instinct, to deposit their eggs in such places as would afford the necessary food to the young and imperfect progeny during the earliest stages of its existence. The Italian observers thus explained, in the most natural manner, what seemed to be a very puzzling circumstance, viz., that these worms, which always showed themselves in great numbers in decomposing animal substances, were not to be found in any other situations. The species seemed to be confined to putrefying substances; and as they often appeared under circumstances which precluded the idea of their having been transferred from other collections of decomposing matter, it was not easy to understand how they could have originated by the ordinary mode of generation. Redi and Valisnieri, however, demonstrated that they were the progeny of perfect insects; and that the *species* was not in reality confined to decomposing substances, but existed elsewhere, though in a different form.

The existence of the infusorial animalcula, again, seemed for a time explicable only on the supposition that they were produced spontaneously in the animal or vegetable infusions which they inhabited. But this supposition has been set entirely at rest since the experiments of Schultze, at Berlin, in 1837, proved that though the production of infusoria was almost invariable when the infusion was kept at the proper temperature and exposed to the access of atmospheric air,—yet that the animalcules were not generated if sufficient care were taken to preclude all possibility of living germs being introduced into the infusion from without. We take it, therefore, not even the most determined advocate of the theory of equivocal generation will place much reliance on the celebrated experiment of the production of the *acarus Crossii* from the continued action of galvano-electricity on a chrystalizable saline solution; — particularly as the acari so produced possessed generative organs, and the females, soon after their appearance, provided for a continuance of the species by an abundant production of ova. The chances of the accidental introduction of living germs into the machine during the course of so long-continued an experiment are too great to allow any one to remain satisfied without a personal inspection of the apparatus.

The only remaining defence of the spontaneous-generation theory, is the existence of *entozoa;* and it is behind this last, and apparently most impregnable barrier, that its partisans have finally entrenched themselves. The entozoa, like the infusoria, are confined to certain situations. They are never detected out of the living body. Particular species of animals are even infested by particular species of parasites, and by no others. The Taenia solium inhabits the intestines of the human subject, the T. serrata those of the dog, the T. crassicollis those of the cat. In the same individual, even, different organs are occupied by different entozoa. We must look for the Trichocephalus dispar in the cœcum, for the Strongylus gigas in the kidney, and for the Distoma hepaticum in the liver. "These facts," it has been said,* "seem to show that some extremely local concurrence of circumstances is essential to the production of the several entozoa." But it is very easy to see that these strictly local conditions may be not at all necessary for the *production*, but only for the *development* of the entozoa. It is certainly no more surprising that one species of Ascaris should inhabit the large, and another the small intestine, than that the Lobelia inflata should grow only in dry pastures and the Lobelia cardinalis by the margin of meadow-brooks. The Lichens flourish on the exposed surfaces of rocks and stone walls; while the Fungi vegetate in darkness and moisture, on the decaying trunks of dead trees. Yet no one imagines these vegetables to be spontaneously generated from the soil which they inhabit. The fact is simply this: that if the animal or vegetable germ be deposited in a locality which affords the conditions necessary for its development, it becomes developed; otherwise not. The grains of wheat which had remained for centuries, wrapped up in the cerements of Egyptian mummies germinated freely when exposed, in an appropriate soil, to the influences of light, air, warmth and moisture. The circumstance, therefore, that particular parasites are confined to particular localities

* Stillé's Pathology, Philadelphia, 1848, p. 473.

presents no greater difficulty as to their mode of reproduction, than the same fact regarding other animal and vegetable organisms.

Every articulation of the Taenia solium contains, when in a state of maturity, many thousands of ova; all of which are necessarily expelled from the body when the articulation drops off. Now though the chances are enormous against any particular one of these ova being accidentally transported into the intestinal canal of another individual, it is easy to see that there are many causes in operation by which *some* of them might be so transported. By far the greater number undoubtedly perish, from not meeting with the conditions necessary to their development. One in a thousand, or, perhaps, one in a million, is accidentally introduced into the body of another individual, and consequently becomes developed into a perfect animal.

The greatest difficulty, however, was presented by the *encysted entozoa*, which are not only confined to particular organs, like other parasites, but which are also destitute of any generative apparatus. Now a species which is destitute of generative organs evidently cannot reproduce itself; and the encysted entozoa have therefore been regarded as presenting at least one undoubted instance of equivocal generation, i. e., a progeny without parents. The fact that no similar animals were found external to the body might have been got over; but that those existing as parasites in the parenchyma of living organs were themselves destitute of any generative apparatus, seemed to exclude the idea that they had been produced by the ordinary modes of propagation.

It is strange that those who advocated so strenuously the doctrine of equivocal generation could not see that this circumstance might be explained in the same way with the production of maggots in putrefying meat; a point which had been settled so long ago by Redi and Valisnieri. These maggots differ in structure from their parents because they are as yet incomplete. They may be considered, to some extent, as still in a fœtal condition. For the same reason the generative organs are not yet developed. The larva is incapable of reproducing itself as a larva. But after it has passed through the natural transformations, and its organization is completed, a sexual apparatus appears, and the *species*, though not every individual belonging to it, is found to be perfect. Now, by watching the growth of any one individual, from the egg to the state of a complete insect, we get a history of its transformations, and comprehend that the animal may be destitute of sexual organs at one period of its existence, and provided with them at another. But if the conditions necessary to the later stages of development are wanting, the larva will not be transformed into an insect, but will remain a larva; and, of course, so long as these conditions are wanting, so long will the sexual organs remain absent. And this is precisely the case with the encysted entozoa. They bear very much the same relation to the Tænia as the undeveloped larva to the perfect insect. It is, perhaps, unnecessary to remind our readers that the Tænia is not now considered as a single animal, but as a colony of animals; every articulation being a distinct individual. These articulations are multiplied by a process of budding, which takes place just behind the "head," or most anterior individual of the colony. As new ones appear, those which were previously produced are pushed farther and farther from the "head;" so that the oldest articulations are those situated at the posterior extremity of the chain. The young and imperfect individuals compose the "neck" of the Tænia. They are, as yet, without sexual organs; but as they increase in size, and are gradually removed farther from the head, they become provided with a sexual apparatus, each articulation containing both male and female organs; so that the posterior portion of the chain is composed of completely developed hermaphrodite individuals. As these individuals arrive successively at maturity, they become detached from the chain, and pass out of the intestine with the fæces, after which the ova are probably set free by the death and decomposition of the parts which enclose them. A portion, then, of every "tape-worm" is destitute of reproductive organs, and yet it has itself undoubtedly been produced from ova, and will hereafter, if circumstances are favorable, produce ova in its turn.

Now the following experiments, by Prof. Von Siebold, demonstrate the very important point that those parasites which have been regarded as incapable of reproduction as a species, are really incapable of it only as individuals, because they have been prevented from arriving at their mature condition; and that the sexless encysted entozoa are in reality only undeveloped or diseased Tæniæ. We have ascertained by personal inquiry, what might have been anticipated from the previous reputation of the observer, that both the experiments, and the results derived from them, are regarded by scientific men in Germany, as entitled to complete confidence. The following account of the experiments is translated from a report, in the "Silesian Times," of the Transactions of the "Silesian Association for National Instruction, Scientific Department, Session of July 7th, 1852:"

*Report.*

Professor Von Siebold made a report on the experiments which were undertaken some months previously, in the Physiological Institute, under his direction, for the purpose of showing the possibility of a transformation of the cystic parasites into tape-worms. He had already, in the year 1844, in the second volume of the Encyclopœdia of Physiology, expressed the opinion that the parasitic cysticercus (C. fasciolaris,) found in the liver of rats and mice, was nothing else than an abnormal, dropsical tape-worm; and that it was, in reality, identical with the tape-worm of the cat (Tænia crassicollis.) He maintained further that the Cysticercus fasciolaris, like all cystic worms, was invariably destitute of sexual organs, and could not multiply its species by generation, unless it were transferred to a favorable locality, where it might lose its dropsical condition and develope its sexual organs. These changes actually take place when a rat or a mouse, with a Cysticercus fasciolaris in its liver, is devoured by a cat. The parenchyma of the liver, according to Siebold, is digested in the stomach of the cat; but not so the entozoon. The parasitic animal loses only its dropsical appendage, and passes, with the digested food, from the stomach of the cat into the small intestine. It then finds itself in a favorable locality, and becomes developed into a perfect tape-worm, with articulations and sexual organs (Tænia crassicollis.) This idea had been first suggested to Prof. Siebold by the perfect resemblance between the cephalic extremity of the Cysticercus fasciolaris and that of the Tænia crassicollis; and by the fact that there are often found, in the intestine of the cat, several specimens of the T. crassicollis, in different stages of development. His opinion was adopted by many naturalists, but its correctness had also been called in question by others. Some years ago Dr. Kuchenmeister, of Zittau, had made use of the Cysticercus pisiformis, a species of encysted parasite very common in the peritoneum of hares and rabbits, for a series of experiments in which he caused these parasites to be swallowed by dogs and cats; in the expectation that they would become developed into tape-worms in the intestine of these animals. The trial sueceeded perfectly with dogs; and the same thing that Prof. Siebold had previously inferred from a comparison of the Cysticercus fasciolaris of rats and mice with the Tænia crassicollis of the cat, would seem to have been definitely established by these experiments of Kuchenmeister. But Kuchenmeister's experiments, and the conclusions he drew from them, did not prove satisfactory either to naturalists or medical men. He had committed the error of publishing his investigations before they could properly be considered as terminated. He was consequently obliged, in the various communications which he published on the subject, one after another, in the medical journals of northern and southern Germany, to correct many of his former statements, and even to retract some of them; and he entangled himself, finally, in so many contradictions that it is to be feared the doctrine of a close relation between cysticerci and tape-worms was rather retarded than advanced by his activity; particularly as he himself several times acknowledged that he was not sufficiently familiar with the study of intestinal worms to distinguish them with certainty. Such a confession certainly was not calculated to increase the confidence of naturalists in his experiments. He also exhibited his incapacity to distinguish the entozoa by giving, in succession, several different names to the newly-produced tape-worm, which he described at first as the "Tænia crassiceps" of the fox, afterward as the "Tænia serrata" of the dog, and finally as an entirely new species, under the name of the "Tænia pisiformis." Prof. Siebold then determined to undertake himself similar experiments. They were tried principally on young dogs, not only with the Cysticercus pisiformis, but also with the C. cellulosa, C. tenuicollis, Cænurus cerebralis, and Echinococcus veterinorum.

The following results were obtained from the experiments with the Cysticercus piriformis. These entozoa, which are usually about the size of a pea, were given to young dogs, mixed with milk, still enclosed in their peritoneal cysts, and in quantities varying from thirty to sixty. The dogs were afterward killed with chloroform, at various intervals of time, and the contents of the stomach and intestine being carefully examined, the entozoa were readily discovered in various stages of development. Two hours after being swallowed, they were almost all found still in the cavity of the stomach. The cysts, however, which had enclosed them, were generally digested and destroyed, and at the same time, the greater number were not only freed from their envelope, but had also lost the vesicular portion of their posterior extremity. This vesicle was either entirely digested or else hung in shreds attached to the end of the body. All the entozoa, which were found in the stomach, whether they had lost the vesicular appendage or not, invariably had the head and neck drawn back into the body.

When the dogs were killed after an interval of three hours, no entozoa were found remaining in the stomach. They had all passed, together with the digested food, into the small intestine. Their cysts and vesicular appendages had all been destroyed by the digestive processes in the stomach; but the head and neck were again, without exception, protruded, and the body, which had been before contracted, was stretched out longitudinally. In all of them were to be seen marks of injury at the posterior extremity of the body, where the vesicular appendage had been attached. When the dogs were suffered to live several days after the commencement of the experiment, the entozoa were found to have considerably increased in size. The largest had attained the length of three inches, the smallest that of one inch. The body, which had previously shown only transverse wrinkles, now exhibited very plainly articulations in its central portion. The posterior portion was still wrinkled transversely, and the lacerated spot at its extremity had assumed the appearance of a cicatrix.

After twenty to twenty-five days, the entozoa were already several inches long, and perfectly articulated quite to their posterior extremity, where the cicatrix was still evident; and on the posterior articulations were to be discovered traces of a sexual apparatus.

After eight weeks the cysticerci in the intestine of one of the dogs had attained a length of many inches. The largest were thirty-six to thirty-nine inches in length, and their posterior articulations were provided with a perfectly developed sexual apparatus, and contained many mature ova. Several of those a yard long had already thrown off their posterior articulations, with their mature sexual products. Von Siebold was now able to recognise, in this tape-worm, developed out of the Cysticercus pisiformis, the Tænia serrata of the dog. The cephalic extremity, the form of the articulations, the structure of the generative organs, and particularly the mature ova of this tape-worm, corresponded, in the most perfect manner, with the same parts in the Tænia serrata. There was no longer any doubt that the Cysticercus pisiformis of the hare and rabbit bore the same relation to the Tænia serrata of the dog, as the Cysticercus fasciolaris of rats and mice to the Tænia crassicollis of the cat. Furthermore, the T. serrata is rarely met with in the intestines of parlor and house dogs, but is, on the contrary, very abundant in hunting dogs; no doubt because the latter are often allowed to devour the entrails of hares killed in the chase, swallow at the same time the Cysticercus, and so become infested with the Tænia; a circumstance which would naturally be less frequent with parlor and house dogs.

Although Siebold's experiments with the other species of encysted entozoa, mentioned above, were not entirely finished, he had yet carried them so far with the "Cœnurus cerebralis" as to convince himself that this worm, also, which is so much dreaded by sheep-breeders, becomes developed, in the alimentary canal of the dog, into a tape-worm. The tape-worm produced from this parasite had not yet, in Siebold's experiments, become developed to the stage of sexual maturity; so that he was still unable to determine with certainty its species. He hoped, however, by means of continued trial, to produce from the Cœnurus cerebralis perfectly mature Tæniæ; so that he might be able, after distinguishing their species, to determine what animal it is in whose intestine the sexless Cœnurus cerebralis becomes developed into a tape-worm with mature sexual apparatus. He will then, probably, have it in his power to give agriculturalists some hints how to prevent the development of this parasite in the brain of the sheep. For he is convinced that the encysted entozoa do not originate by spontaneous generation, but are produced from the microscopic ova of the tape-worms of certain carniverous animals, which are introduced by accident into the bodies of rodentia and ruminantia. Here they are not developed into tape-worms; but degenerate into encysted worms, which exert a more or less injurious influence on the life of the animal, according to the importance of the organ in which they have taken up their residence and at the expense of which they live.

The experiments which have been commenced with the Echinocoecus veterinorum have already shown that this parasite is also to be considered as a tape-worm. The progeny of this destructive entozoon are produced, as is well known, in great numbers, by a process of budding from its inner surface. These were given in spoonfuls to young dogs, and in a few days afterward thousands of exceedingly small tape-worms were discovered, fastened by their four suckers to the mucous membrane of the small intestine. The bodies of these tape-worms consisted of only three divisions, viz., a head and neck for the first division, then a small articulation, and finally a longer one at the extremity. In both of these articulations the sexual organs had already begun to show themselves. They were not yet, however, so far developed that the worms could be considered as in a state of maturity, or their species accurately determined. It is Prof. Siebold's intention to continue these experiments; and he hopes, at some future time, to communicate their results to the Association.

